# Clinical characteristics and short-term outcomes of acute pancreatitis among patients with COVID-19

**DOI:** 10.1186/s40001-023-01252-x

**Published:** 2023-08-16

**Authors:** Jinchang Zhang, De Luo, Maoji Kang, Bo Li, Song Su

**Affiliations:** Department of General Surgery (Hepatobiliary Surgery), The Affiliated Hospital of Southwest Medical University, Luzhou, 646000 Sichuan China

**Keywords:** SARS-CoV-2, COVID-19, Acute pancreatitis, COVID syndrome

## Abstract

**Objective:**

The existing literature on the combination of acute pancreatitis (AP) and COVID-19 is scarce. The objective of our study is to compare the clinical outcomes and occurrence of long COVID syndrome in AP patients with and without COVID-19, while investigating the potential impact of COVID-19 on the severity, mortality rate, and long COVID syndrome in these patients.

**Methods:**

This retrospective, observational study was conducted at a single center. It included patients aged 18 years and above who were diagnosed with AP during the pandemic. Patients were categorized into two groups based on the results of RT-qPCR testing: the SARS-CoV-2-positive group and the SARS-CoV-2-negative group. The study aimed to compare the severity of AP, mortality rate, and occurrence of long COVID syndrome between these two groups.

**Result:**

A retrospective review was conducted on 122 patients diagnosed with acute pancreatitis between December 1, 2022, and January 31, 2023. Out of these patients, 100 were included in the study. The analysis revealed no significant differences in mortality rate, severity, and sequelae between AP patients with COVID-19 and those without COVID-19 (*p* > 0.005). However, a statistically significant difference was observed in the occurrence of long COVID syndrome, specifically in the presence of cough (*p* = 0.04).

**Conclusion:**

This study demonstrates that the presence of COVID-19 in patients with pancreatitis does not lead to an increase in the mortality and severity rate of pancreatitis.

## Background

Acute pancreatitis (AP) is a common gastrointestinal disease that typically requires hospitalization [1]. Severe AP accompanied by organ failure is closely associated with poor patient prognosis and increased risk of mortality [2, 3].

In late December 2019, a novel respiratory syndrome emerged in Wuhan, China. Subsequent testing confirmed it to be severe acute respiratory syndrome coronavirus 2 (SARS-CoV-2). A few months later, the World Health Organization declared it a major public health concern. The clinical manifestations of COVID-19, the disease caused by SARS-CoV-2, are wide-ranging and include fever, cough, and dyspnea [4]. Additionally, less common symptoms encompass loss of smell and taste, gastrointestinal symptoms, headache, and rash [5–8]. Since the onset of the pandemic, there has been a growing body of literature focused on exploring treatment strategies and reducing mortality associated with COVID-19 [9–11]. As of January 2023, there have been more than 850 million reported cases of COVID-19 worldwide, resulting in over 6.6 million deaths [12]. However, COVID-19 remains a substantial global health threat. Apart from its mortality rate, COVID-19 presents an unparalleled risk to the mental well-being of individuals worldwide. Numerous patients with COVID-19 encounter emotional disorders and exhibit symptoms of depression [13–15].

Although strict epidemic prevention measures have led to a decline in the number of COVID-19 infections, China made adjustments to its epidemic prevention policy on December 7, 2022, resulting in a surge in cases from December 2022 to January 2023. During this period, the primary variant circulating in China was Omicron [16]. Previous studies have shown a significantly increased risk of adverse outcomes and a mortality rate close to 20% in patients with concurrent AP and COVID-19 [17]. Numerous studies have reported an increase in pancreatic enzymes in patients with COVID-19 [18], indicating potential pancreatic damage associated with the disease. However, with the increasing coverage of vaccines, the mortality rate of COVID-19 patients continues to decline [19]. The mortality rate of patients with concurrent acute pancreatitis and COVID-19 may undergo changes in the near future. However, there has been a scarcity of articles in this research area over the past 2 years, highlighting the need for further investigation to address this knowledge gap.

Therefore, we conducted a retrospective review of all hospitalized patients with AP in our hospital, comparing the clinical outcomes and short-term outcomes between patients with COVID-19-associated AP and those with SARS-CoV-2-negative AP.

## Materials and methods

### Study design

This is a single-center, retrospective, and observational study.

### Population and study time

We screened patients who visited the emergency department of our hospital from December 1, 2022, to January 31, 2023, using the hospital electronic system.

### Inclusion and exclusion criteria

The inclusion criteria were all patients with acute pancreatitis (AP) in the entire hospital, with available reverse transcriptase real-time (RT-qPCR) results, laboratory tests, and imaging results. If a patient had multiple admissions due to AP during the study period, only the last admission was considered for eligibility.

### Setting

General population in Luzhou City, Sichuan Province.

### Assessments

The clinical diagnosis of COVID-19 is primarily based on epidemiological history, clinical manifestations, and laboratory testing methods. The laboratory testing method involves using an oropharyngeal swab sample for RT-qPCR [20]. Based on the RT-qPCR test results, patients are categorized into positive and negative groups.

#### Data collection

We analyzed the mortality rates of COVID-19 patients as reported in relevant studies [21]. In order to ensure sufficient statistical power and precision, we excluded studies that did not meet the predetermined sample size threshold of 81. This threshold was calculated using PASS 15 software, with a confidence level of 0.95, a confidence interval width of 0.227, and an assumed proportion of 0.454. The purpose of this sample size threshold was to ensure that the included studies had enough power to achieve a margin of error of at least 0.05 on the estimated prevalence. Considering an estimated loss of follow-up rate of 10%, it is recommended that the final sample size should not be less than 89. This adjustment accounts for potential attrition and ensures that an adequate number of participants remain in the study for reliable analysis.

Upon admission, clinical laboratory tests including white blood cell (WBC) count, lymphocyte (Lym) count, neutrophil (Neu) count, neutrophil-to-lymphocyte ratio (NLR), hematocrit (HCT), red cell distribution width (RDW), platelet (PLT) count, C-reactive protein (CRP), D-dimer levels, and imaging findings are recorded (Table 1). Patient history regarding alcohol consumption, family history (genetic diseases), laboratory tests (hyperlipidemia), and diagnostic procedures such as computed tomography (CT), endoscopic ultrasound, magnetic resonance cholangiopancreatography (MRCP), and endoscopic retrograde cholangiopancreatography (ERCP) are examined to determine the etiology of the patient's condition. Patients in whom no cause is identified after evaluation are documented as having “unknown etiology”.

**Table 1:** Comparison of laboratory parameters between positive and negative patients with COVID-19 on the first day of admission

Variables	Positive(*n* = 26)	Negative(*n* = 74)	*p* value
WBC (× 10^9^/L)	10.95 ± 5.45	14.13 ± 7.26	**0.02**
NEU (× 10^9^/L)	9.15 ± 5.67	12.09 ± 6.82	**0.03**
LYM (× 10^9^/L)	1.4 ± 1.22	1.22 ± 0.8	0.48
MONO (× 10^9^/L)	0.9 ± 1.99	0.76 ± 0.52	0.08
EOS (× 10^9^/L)	0.04 ± 0.05	0.07 ± 0.11	0.23
BASO (× 10^9^/L)	0.03 ± 0.04	1.25 ± 10.46	**0.04**
NEU-R (%)	78.72 ± 12.35	82.17 ± 11.94	0.14
LYM-R (%)	15.48 ± 12.78	10.21 ± 7.49	0.08
MONO-R (%)	5.52 ± 3.14	5.54 ± 3.7	0.54
EOS-R (%)	0.49 ± 0.72	0.64 ± 0.92	0.56
BASO-R (%)	0.23 ± 0.2	0.28 ± 0.39	0.40
RBC (× 10^12^/L)	4.6 ± 0.93	4.44 ± 0.75	0.39
HGB (g/L)	139.35 ± 23.82	143.03 ± 29.22	0.57
HCT	0.4 ± 0.06	0.4 ± 0.07	0.90
PLT (× 10^9^/L)	198.23 ± 74.73	217.92 ± 116.57	0.60
PT (s)	13.49 ± 1.58	13.36 ± 1.33	0.70
APTT (s)	30.13 ± 4.1	30.47 ± 6.49	0.49
TT (s)	17.32 ± 1.56	17.19 ± 2.14	0.40
ALT (U/L)	106.86 ± 197.63	78.89 ± 129.13	0.88
AST (U/L)	120.58 ± 274.38	92.71 ± 155.94	0.42
TBIL (μmol/L)	21.89 ± 24.81	32.43 ± 58.49	0.33
TC (mmol/L)	6.84 ± 5.23	7.23 ± 5.07	0.28
TG (mmol/L)	6.63 ± 8.17	7.41 ± 7.7	0.32
HDLC (mmol/L)	1.3 ± 1.06	1.14 ± 0.7	0.47
LDLC (mmol/L)	2.78 ± 1.07	3.25 ± 1.45	0.18
Crea (μmol/L)	76.22 ± 46.92	73.5 ± 56.59	0.86
GFR (mL/min)	102.33 ± 33.25	103.62 ± 24.82	0.70
AMY (U/L)	608.53 ± 776.22	473.9 ± 682.32	0.60
PAMY (U/L)	510.77 ± 690.01	387.32 ± 579.75	0.65
LPS (U/L)	734.93 ± 880.1	706.84 ± 938.63	1.0
K (mmol/L)	3.96 ± 0.49	4.01 ± 0.64	0.90
Na (mmol/L)	138.77 ± 4.99	135.51 ± 6.33	**0.03**
Cl (mmol/L)	105.35 ± 6.34	103.46 ± 5.68	0.22
Ca (mmol/L)	2.33 ± 0.22	2.28 ± 0.25	0.41
NT-proBNP (ng/L)	557.1 ± 630.88	289.64 ± 462.1	0.06
PCT (ng/mL)	3.47 ± 9.53	1.38 ± 3.56	0.92
IVGLU (mmol/L)	9.2 ± 4.36	10.07 ± 6.35	0.70
FBGLU (mmol/L)	9.55 ± 3.66	11.02 ± 5.9	0.45

P value less than 0.05 is represented in bold

Summary statistics were presented as number (%) for categorical variables and as mean ± SD for continuous variables. A two-sided *p* value of less than 0.05 was considered statistically significant

*WBC* White blood cell, *NEU* neutrophil, *LYM* lymphocyte, *MONO* monocyte, *EOS* eosinophils, *BASO* basophils, *NEU-R* neutrophil rate, *LYM-R* lymphocyte rate, *MONO-R* monocyte rate, *EOS-R* eosinophil cell rate, *BASO-R* basophil cell rate, *RBC* red blood cell, *HGB* hemoglobin, *HCT* hematocrit, *PLT* platelet, *PT* prothrombin time, *APTT* activated partial thrombin time, *TT* thrombin time, *ALT* alanine aminotransferase, *AST* aspartate aminotransferase, *TBIL* total bilirubin, *TC* total cholesterol, *TG* triglyceride, *HDLC* high-density lipoprotein cholesterol, *LDLC* low-density lipoprotein cholesterol, *Crea* creatinine, *GFR* glomerular filtration rate, *AMY* amylase, *PAMY* pancreatic amylase, *LPS* lipase, *NT-proBNP* N-terminal pro brain natriuretic peptide, *PCT* procalcitonin, *IVGLU* intravenous glucose, *FBGLU* fingertip blood glucose

Patients who underwent interventional treatments (ERCP, PTC), surgical interventions (such as cholecystectomy), and pharmacological treatment for AP are documented. The length of hospital stay (intensive care/service), AP-related complications, and mortality rate are recorded. During the month of March 2023, a telephone follow-up was conducted for all patients to inquire about the presence of long COVID syndrome. Refusal to participate in the telephone follow-up or patient refusal to answer relevant questions is considered as loss to follow-up.

#### Research definition

The diagnosis of acute pancreatitis (AP) is based on the revised Atlanta criteria [22]. Comorbidities are classified using the Charlson Comorbidity Index (CCI) [23]. Long COVID syndrome is defined as the presence of persistent symptoms and/or delayed or long-term complications lasting for more than 4 weeks after symptom onset [24]. The severity of AP is determined using the Bedside Index for Severity in Acute Pancreatitis (BISAP) score [25]. A BISAP score of ≥ 3 indicates severe AP, while a score of ≤ 2 indicates mild AP [26]. We also utilize the Balthazar CT Severity Index (CTSI) to assess the severity of AP, with severity categorized as mild (0–3 points) or moderate to severe (4–10 points) [27].

Organ failure is defined as a Marshall score [28] of ≥ 2 in one or more of the three organ systems (respiratory, renal, and cardiovascular) initially described. The organ failure score is calculated based on the most extreme laboratory values or clinical measurements within the first 72 h prior to hospital admission for all patients. The duration of organ failure is defined as transient (≤ 48 h) or persistent (> 48 h).

#### Statistical analysis

The research data were evaluated using IBM SPSS Statistics version 27. The normality of quantitative data was analyzed using the one-sample Kolmogorov–Smirnov test, which helped determine whether to use parametric or non-parametric tests. Descriptive statistics were computed for frequency and percentage distributions, as well as for continuous variables, including mean, standard deviation, median, minimum, and maximum values. For comparing categorical variables between groups, the Pearson chi-square test and Fisher’s exact test were employed. The Mann–Whitney *U* test was used to analyze differences between laboratory values and SARS-CoV-2 test results. Additionally, laboratory parameters were evaluated by comparing SARS-CoV-2 positive and negative patients with acute pancreatitis. A significance level of *p* < 0.05 was considered statistically significant.

### Ethical issues

This retrospective and observational study did not provide any additional interventions to the included patients. The retrospective study design has been approved by the Clinical Trial Ethics Committee of the Affiliated Hospital of Southwest Medical University, China (Approval No: KY2023190).

This study was conducted in accordance with the Helsinki Declaration. A written informed consent form has been exempted by the ethics review committee, because we only retrospectively extracted anonymous data without any protected information. Each patient participating in this study was assigned a unique identifier, and all anonymous patient data were recorded and analyzed in relation to this identifier.

## Results

We conducted a retrospective analysis of 122 patients diagnosed with acute pancreatitis (AP) between December 1, 2022, and January 31, 2023. Among these patients, there were 100 individuals who met the inclusion criteria and had sufficient data recorded. The positivity rate for SARS-CoV-2 was found to be 26%. Among the included patients, 47.6% were male, with a mean age of 54.56 ± 16.90 years, while 52.4% were female, with a mean age of 55.24 ± 19.00 years.

The patients were divided into two groups: the SARS-CoV-2 positive group (*n* = 26) and the SARS-CoV-2 negative groups (*n* = 74). There were no significant differences in baseline characteristics between the two groups (Table 2). In terms of comorbidity distribution, there was no significant difference in the proportion of patients with a CCI score ≥ 5 between the positive and negative groups (Table 3). When comparing short-term organ failure between the two groups, there was no statistically significant difference (*p* = 0.69). Similarly, there was no statistically significant difference in the occurrence of persistent organ failure between the positive group (3.85%) and the negative group (4.05%) (*p* = 1.0). The BISAP score and Balthazar CT index also showed no statistically significant difference (*p* = 0.88, *p* = 0.43), indicating no significant difference in disease severity between the two groups. After analyzing the mortality rate among hospitalized patients, it was found that the mortality rate was 3.85% for the positive group and 4.05% for the negative group, with no statistically significant difference (*p* = 1.0).

**Table 2:** Demographic characteristics of patients

Variables	Category	Positive (*n* = 26)	Negative (*n* = 74)	*p* value
Age	*n* (mean ± SD)	46.46 ± 17.3	48.8 ± 12.8	0.53
Gender	Male/female	15/11	46/28	0.69
BMI	Mean ± SD	25.95 ± 3.54	25.49 ± 4.37	0.50
Smoke	*n* (%)	7 (26.92)	29 (39.19)	0.26
Alcohol	*n* (%)	8 (30.77)	26 (35.14)	0.69
Hypertension	*n* (%)	6 (23.08)	15 (20.27)	0.76
Diabetes	*n* (%)	8 (30.77)	24 (32.43)	0.88
Etiology	Hyperlipidemia, *n* (%)	7 (26.92)	21 (28.38)	0.97
	Gallstone, *n* (%)	8 (30.77)	21 (28.38)	
	Alcohol, *n* (%)	5 (19.23)	17 (22.97)	
	Others, *n* (%)	6 (23.08)	15 (20.27)	
Neocoronal vaccine	*n* (%)	19 (95.0)^a^	58 (90.63)^a^	1.0

Summary statistics were presented as number (%) for categorical variables and as mean ± SD for continuous variables. A two-sided *p* value of less than 0.05 was considered statistically significant

*BMI* body mass index (weight in kilograms divided by height in meters squared)

^a^During hospitalization, there was no information about patient vaccination, so this information was obtained through follow-up. The percentage is the number of patients vaccinated compared to the total number of follow-up patients

**Table 3:** COVID-19 positive–negative status and distribution of variables

Variables	Category	Positive (*n* = 26)	Negative (*n* = 74)	*p* value
CCI score	1–2, *n* (%)	24 (92.3)	69 (93.24)	1.0
	3–4, *n* (%)	1 (3.84)	3 (4.05)	
	≥ 5, *n* (%)	1 (3.84)	2 (2.7)	
BISAP score	< 3, *n* (%)	20 (76.92)	58 (78.38)	0.88
	≥ 3, *n* (%)	6 (23.08)	16 (21.62)	
Organ failure	None, *n* (%)	24 (92.3)	64 (86.49)	0.69
	Single-organ failure, *n* (%)	2 (7.69)	6 (8.11)	
	Double-organ failure, *n* (%)	0 (0)	3 (4.05)	
	Triple organ failure, *n* (%)	1 (3.84)	1 (1.35)	
Persistent organ failure, *n* (%)		1 (3.85)	3 (4.05)	1.0
Mortality, n (%)		1 (3.85)	3 (4.05)	1.0
Balthazar CT index	< 4, *n* (%)	15 (57.69)	36 (48.65)	0.43
	≥ 4, *n* (%)	11 (42.31)	38 (51.35)	
ICU	*n* (%)	2 (7.69)	3 (4.05)	0.83
Hospitalization length	Mean ± SD	11.69 ± 8.79	9.75 ± 4.79	0.51

Summary statistics were presented as number (%) for categorical variables and as mean ± SD for continuous variables. A two-sided *p* value of less than 0.05 was considered statistically significant

*CCI* Charlson Comorbidity Index, *BISAP score* Bedside index for severity in acute pancreatitis, *ICU* Intensive care unit, COVID-19, the novel coronavirus disease 2019

A telephone follow-up was conducted for all AP patients (Table 4), and it was found that 16 individuals were lost to follow-up, resulting in a loss to follow-up rate of 16%. Regarding long COVID syndrome, only coughing showed a statistically significant difference (*p* = 0.04).

**Table 4:** Follow-up table after 1 month for positive and negative patients with COVID-19

Variables	Category	Positive (*n* = 20)	Negative (*n* = 64)	*p* value
Abdominal pain	*n* (%)	6 (30.0)	8 (12.50)	0.09
Recurrence^a^	*n* (%)	3 (15.0)	3 (4.69)	0.14
Poor appetite	*n* (%)	3 (15.0)	8 (12.50)	0.72
Nausea and vomiting	*n* (%)	3 (15.0)	3 (4.69)	0.14
Diarrhea	*n* (%)	2 (10.0)	6 (9.38)	1.0
Constipation	*n* (%)	1 (5.0)	2 (3.13)	0.56
Loss of weight	*n* (%)	3 (15.0)	24 (37.50)	0.10
Tired	*n* (%)	7 (35.0)	17 (26.56)	0.57
Cough	*n* (%)	3 (15.0)	1 (1.56)	**0.04**
Headache	*n* (%)	2 (10.0)	1 (1.56)	0.14
Dizziness	*n* (%)	2 (10.0)	3 (4.69)	0.59
Chest pain	*n* (%)	2 (10.0)	1 (1.56)	0.14
Myalgia	*n* (%)	1 (5.0)	4 (6.25)	0.66
Palpitate	*n* (%)	3 (15.0)	4 (6.25)	0.35
Dyspnea	*n* (%)	1 (5.0)	0	0.24
Fever	*n* (%)	1 (5.0)	0	0.24
Memory loss	*n* (%)	1(5.0)	0	0.42
Anxiety	*n* (%)	3 (15.0)	6 (9.38)	0.44
Sleep disorder	*n* (%)	4 (20.0)	8 (12.50)	0.47

P value less than 0.05 is represented in bold

Summary statistics were presented as number (%) for categorical variables and as mean ± SD for continuous variables. A two-sided *p* value of less than 0.05 was considered statistically significant

^a^It refers to the recurrence of pancreatitis within 1 month Sequelae

## Discussion

We present here the clinical and short-term outcomes of patients with acute pancreatitis (AP) admitted during the outbreak of the novel coronavirus pneumonia in China. We found that SARS-CoV-2-positive AP patients had similar mortality rates and disease severity compared to SARS-CoV-2-negative AP patients, with no statistically significant differences observed across various scoring systems. Follow-up at 1–2 months revealed that AP patients who tested positive for SARS-CoV-2 were more likely to experience cough symptoms (*p* = 0.04). Although there was a higher proportion of positive cases reporting abdominal pain, the difference was not statistically significant (*p* = 0.088).

A meta-analysis has indicated a high risk of adverse outcomes, with a mortality rate close to 20%, in patients with both AP and COVID-19 [17]. However, in our study, we found no statistically significant difference in the mortality rates between AP patients with and without COVID-19 (*p* = 1.0). The mortality rate among AP patients with COVID-19 was 3.85%, which is considerably lower than the mortality rate reported in the literature.

The lower mortality rate observed in our study may be attributed to the fact that a majority of patients (91.7%) received vaccination. Research has shown that vaccines provide a significant level of protection against severe COVID-19, reducing hospitalization rates and mortality [19, 29]. As of April 7, 2021, a total of 710 million vaccine doses have been administered globally, with at least one dose of an approved vaccine given to 5% of the world’s population [30]. The studies included in the article by Yang et al. [17] were published between 2020 and 2021, with most of the patients being admitted in 2020. Therefore, it can be inferred that the vaccine coverage among the patients included in Yang et al.'s article was extremely low, which could explain the lower mortality rate observed in our patients.

The potential of SARS-CoV-2 to induce acute pancreatitis remains unclear [31–34]. Indeed, there is evidence suggesting that SARS-CoV-2 has the ability to infect the pancreas and induce pancreatic injury [18, 35–38]. According to our study, there was no significant difference in disease severity between the two groups as indicated by the BISAP score and Balthazar CT index. This may be due to the limited extent of pancreatic damage caused by the current strain of SARS-CoV-2, resulting in no statistically significant difference in disease severity and mortality rates between the two groups.

In our comparison of the initial laboratory test results between SARS-CoV-2-positive and -negative patients on the day of admission, we found statistically significant differences (*p* < 0.05) in the counts of white blood cells, neutrophils, and eosinophils, while other results showed no statistically significant differences (*p* > 0.05). These differences may be attributed to the viral infection.

After a short-term follow-up of 1 month post-discharge, we found that the most commonly reported symptoms of long COVID syndrome among SARS-CoV-2-positive patients were fatigue (35%) followed by abdominal pain (30%). Although both symptoms were relatively high in proportion compared to SARS-CoV-2-negative patients, there was no statistically significant difference (*p* > 0.05). The only symptom that showed a statistically significant difference was cough (*p* = 0.04). The prevalence of cough in our COVID-19 patients after discharge (15%) was similar to what has been reported in other studies [39]. Research has shown that vaccinated individuals and those infected with the Omicron variant have a lower risk of developing long COVID syndrome [40]. This may explain why there was no difference in long COVID syndrome among our patients.

Additionally, studies have reported that even post-infection vaccination can alleviate long COVID syndrome [41]. Therefore, it is recommended that patients who have not received the vaccine after infection should consider getting vaccinated to mitigate the symptoms of long COVID syndrome.

## Limitations

As this study is retrospective, there may be selection bias, and the results may not fully reflect the overall outcomes of patients with COVID-19-associated pancreatitis. Our follow-up was conducted via telephone, which introduces the possibility of recall bias. Additionally, a small proportion of patients were lost to follow-up, which could potentially impact the results. The sample size of our study was relatively small, with only 100 patients. This study was conducted at a single center, which may limit the generalizability of the results. The follow-up period was relatively short (1–2 months), and longer term follow-up was not conducted. Long COVID-19 syndrome typically lasts for 4–8 weeks or longer. Therefore, it is possible that some patients who tested negative for SARS-CoV-2 upon admission may have been infected prior to admission or after discharge, which could introduce bias in the follow-up results of long COVID-19 syndrome.

## Conclusion

Our research findings indicate that there is no significant difference in clinical outcomes and short-term prognosis between patients with COVID-19-related pancreatitis and those without SARS-CoV-2 infection. Furthermore, we observed that individuals who have been vaccinated against COVID-19 and subsequently contract COVID-19-related pancreatitis generally do not require additional medical care.

## Data Availability

The datasets utilized and/or analyzed during the present study are accessible from the corresponding author upon reasonable request.
